# One Catheter, Two Chambers: Feasibility and Acute Procedural Outcomes of Dual-Chamber Leadless Pacemaker Implantation with a Single Delivery Catheter

**DOI:** 10.3390/jcm15218664

**Published:** 2026-11-06

**Authors:** Marcin Kowalski, Kaiyu Jia, Elizabeth Rimsky, Sean Kowalski, Samer Saouma, Rina Shah, Philippe Akhrass, Valay Parikh

**Affiliations:** 1 Cardiovascular Institute, Northwell Health, Staten Island University Hospital, New Hyde Park, NY 11040, USA; 2 Department of Internal Medicine, Staten Island University Hospital, Northwell Health, NY 10305, USA

**Keywords:** leadless pacemaker, pacing, bradycardia, pacemaker catheter implant

## Abstract

**Background**: Dual-chamber leadless pacing with the Aveir DR system restores atrioventricular synchrony without transvenous leads. The labeled implant workflow calls for a separate delivery catheter for each device. The second catheter adds cost and adds steps to the procedure, and whether it is actually needed has not been examined in a large series. Objective: To describe the acute procedural feasibility, the rate of delivery-catheter malfunction, and the effect on direct disposable supply cost of implanting both devices of a dual-chamber leadless pacemaker system through one delivery catheter. **Methods**: We reviewed 102 consecutive patients who received a dual-chamber leadless pacemaker at a single center between February 2024 and February 2025, implanted by five operators at that center. Twenty earlier dual-chamber implants performed with two catheters, before the single-catheter workflow was adopted, precede the study period and are not part of this cohort. The right ventricular device was placed first in every case. The same catheter was then reloaded with the atrial device and used for the second implant, and a new catheter was opened only if the first one failed. We recorded acute implant success, catheter malfunction, procedure time, fluoroscopy time, complications through 30 days after implantation, and direct disposable supply cost. **Results**: Both devices were implanted through the original catheter in 100 of 102 patients (98.0%, 95% CI 93.1 to 99.8%). Two patients (2.0%, 95% CI 0.2 to 6.9%) needed a catheter exchange because of a mechanical failure that arose during ventricular deployment; in both, the ventricular device had been recaptured and redeployed with repeated torque beforehand. No catheter failed during atrial implantation. Mean time from sheath insertion to removal was 69 min, and mean fluoroscopy time was 16.0 ± 6.6 min. Two device dislodgements occurred (2.0%, 95% CI 0.2 to 6.9%), one intraprocedural and one a right atrial device that embolized the day after implant; neither was related to the delivery catheter. There was no perforation, pericardial effusion, or access-site complication, and no other complication occurred in any patient through 30 days after implant. Omitting the second catheter lowered direct disposable supply cost by the cost of the second catheter. **Conclusions**: With experienced operators, one delivery catheter completed both implants in 98% of cases and lowered disposable supply cost. The study is single arm aand the number of events is small, so these findings support feasibility, with 30-day freedom from complications of 98.0%, and a supply cost difference.

## 1. Introduction

Dual-chamber cardiac pacing is a cornerstone therapy for conditions like advanced atrioventricular block and sinus node dysfunction. Traditionally, this has been achieved using transvenous dual-chamber pacemakers with leads in the right atrium and ventricle. However, leadless pacemaker (LP) technology has recently emerged as an innovative alternative that eliminates transvenous leads and surgical pockets [1,2].

Recent advances have led to the development of the first dual-chamber leadless pacemaker systems, the Abbott Aveir™ DR. These systems use two LPs—one implanted in the right atrium and one in the right ventricle—to achieve synchronized dual-chamber pacing [1]. Each self-contained device is delivered percutaneously via a catheter into its target chamber, and the two LPs communicate beat-by-beat to coordinate AV timing [3].

Current implantation protocols for dual-chamber LP remain conservative, typically using two separate delivery catheters—one for each device chamber. The standard workflow involves first deploying the ventricular leadless pacemaker and introducing a new delivery catheter to implant the atrial device [4]. This two-catheter approach is endorsed to ensure optimal handling and placement of each device, and it aligns with manufacturer guidelines for the Aveir system [4]. While effective, the need for a second catheter introduces additional procedural steps, equipment, and cost. In an era where procedural efficiency and resource optimization are increasingly important, streamlining the implantation process by reducing such redundancies is an attractive proposition.

An emerging strategy uses a single delivery catheter to sequentially deploy both the ventricular and atrial devices. The objective of this retrospective study is to describe the acute procedural feasibility of the single delivery-catheter dual-chamber pacemaker implantation technique, the rate at which the catheter malfunctioned, and the effect on disposable supply cost.

## 2. Methods

The retrospective, single-center study cohort consisted of consecutive patients who have guideline indication for dual-chamber pacemaker and were implanted with dual-chamber Aveir™ (Abbott Sylmar, CA, USA) leadless pacemaker system at an urban tertiary care hospital (The Staten Island University Hospital, Northwell Health, NY, USA) from 1 February 2024 to 1 February 2025. This study was approved by the local institutional review board committee. The procedures were performed by five experienced operators at a single center. Our center initially began implanting dual-chamber Aveir devices using two catheters for each procedure. After completing 20 procedures, all operators transitioned to using a single delivery catheter per case. Those first 20 two-catheter procedures precede the study period and are not included in the 102 patients reported here, so the analytic cohort consists entirely of intended single-catheter procedures (Figure 1). Procedure time, fluoroscopy time and recapture data were not recorded prospectively for those 20 cases, so no internal two-catheter comparison group is available.

Sequential implantation of both devices with one delivery catheter is not the workflow described in the manufacturer’s instructions for use, which specify withdrawal of the ventricular delivery catheter and loading of the atrial device onto a new catheter [4]. The single-catheter approach was therefore a deviation from the recommended workflow. It was adopted as a physician-directed procedural decision within the operators’ clinical judgment rather than as a study intervention, and both devices were used within their approved indications. The institutional review board approved this retrospective review of records and waived informed consent for the review.

### 2.1. Procedural Details

The procedure was performed in a cardiac electrophysiology lab under fluoroscopic guidance. The implant techniques of dual-chamber Aveir™ had been described previously [5]. Four heparinized saline infusions were prepared for continuous flushing and connected to the delivery catheter. Percutaneous venous access was obtained in the right femoral vein, and a large introducer sheath (Aveir™ Introducer, Abbott Sylmar, CA, USA) was inserted into the femoral vein and flushed with heparinized saline per manufacturer instruction. The ventricular LP was loaded onto the delivery catheter using the provided loading tool. The catheter’s controls were set to the designated loading position (short tether mode), and the LP was docked to the catheter by pulling back on the LP control knob at the back end of the delivery-catheter handle until an audible click was heard. The protective sleeve was advanced to cover the LP’s fixation helix, and the valve bypass tool was inserted into the introducer.

With the right ventricular LP loaded, the delivery catheter was introduced through the femoral sheath and advanced across the tricuspid valve into the right ventricle. The delivery catheter was navigated to the desired implant site in the right ventricle, typically the mid-septal endocardium of the RV septum. A septal position was confirmed by fluoroscopy and injection of contrast to delineate the endocardial border and confirm the target location. The protective sleeve was retracted to expose the ventricular LP’s helix, and optimal sensing and pacing parameters were analyzed. The pacemaker was screwed into the septal myocardium with 1.5 rotations. Once the device was engaged in the tissue, the catheter was put in short tether mode and a fixation test was performed by deflecting the catheter. This confirmed secure fixation in the ventricular wall. While the RV LP remained tethered, intracardiac signals and pacing efficacy were evaluated. If values are suboptimal, the LP was repositioned, and the measurements were repeated until acceptable results were found. Once optimal parameters were found, the device was released and the delivery catheter was retracted. The number of fixation and recapture attempts was not recorded prospectively.

The same delivery catheter was then used for the right atrial LP implant. This differs from the manufacturer’s instructions for use, which specify withdrawal of the ventricular delivery catheter and loading of the atrial device onto a new catheter [4]. After the ventricular device was released, the catheter was withdrawn from the introducer and inspected on the table. The operator examined the distal cap, the protective sleeve and the shaft for kinking, deformation or retained thrombus, confirmed that the handle moved freely between the docking, short tether and long tether positions, and flushed all four lumens with heparinized saline until neither air nor blood returned. Visible thrombus, blood in the tether channel that did not clear with flushing, a handle that would not transition between tether modes, shaft kinking, and any unusual sound or resistance during the ventricular implant were treated as grounds for mandatory exchange rather than reuse. The atrial LP was then prepared and loaded onto the delivery catheter in the same manner as the first device. The loaded atrial LP and the delivery catheter were introduced through the femoral sheath and advanced into the right atrium. Using fluoroscopy in multiple views to improve spatial orientation, the catheter was advanced until the atrial LP was near the endocardial surface at the target site, typically under the right atrial appendage. Contrast was injected through the catheter to verify position relative to atrial wall. Once the catheter was in place, the LP’s protective sleeve was retracted to expose its fixation helix at the tip. Optimal sensing and pacing parameters were analyzed at the chosen site.

The leadless pacemaker was fixed to the right atrial myocardium by engaging its helix into the tissue by rotating 1.5 rotations of the device. Once the device was engaged in the tissue, the catheter was placed in long tether mode. A gentle pull test (tether deflection test) was performed to verify secure fixation.

With the atrial LP in tethered position, pacing capture thresholds and P-wave sensing amplitudes were measured. If the pacing/sensing performance was not within acceptable ranges, the LP was repositioned by adjusting the catheter and helix engagement (repeating positioning and fixation as needed). Once satisfactory position, fixation, and electrical performance were confirmed, the atrial LP was released. The two leadless pacemakers were wirelessly paired and the i2i signal quality and latency was optimized. Procedural success was defined as successful deployment and function of both an atrial and a ventricular leadless pacemaker in the same session using one catheter. When the delivery catheter malfunctioned, it was returned to Abbott for evaluation. Procedure duration was calculated from venous access until the removal of the large introducer sheath at the end of the procedure. Complications were ascertained from the procedure record, the inpatient chart and the 30-day post-implant follow-up records, and from any unscheduled encounter that came to the attention of the implanting service. Complications were assessed through 30 days after implantation.

### 2.2. Statistical Analysis

Study results are presented using descriptive statistics. For continuous variables, the results include number, mean, and SD where pertinent. Categorical variables are presented as the number and percent in each category. Because the study is single arm and has no comparator, no between-group hypothesis testing was performed and no *p* values are reported. The effect of catheter-exchange frequency on supply cost was examined in a deterministic one-way sensitivity analysis across exchange rates from 0% to 20%. All analyses were performed using SPSS 19.0 (SPSS Inc. Chicago, IL, USA).

## 3. Results

Between February 2024 and February 2025, 102 consecutive patients underwent dual-chamber Aveir implantation with an intended single-catheter workflow, and these patients form the analytic cohort. The 20 earlier procedures performed with two catheters preceded adoption of the technique, fall outside the study period, and were not analyzed. No patient in the study period was excluded, and none was lost before discharge. Figure 1 shows the patient flow.

The cohort consisted of 102 patients, average age 77.3 ± 10 years, with 57.8% male. Sinus node dysfunction was the leading reason for pacemaker implants (48%), followed by complete heart block (20.6%), second or high-degree AV block (18.6%), and tachy-brady syndrome (12.7%). Hypertension was the most common comorbidity (82.4%), with coronary artery disease (35.3%), diabetes mellitus (42.2%), and atrial fibrillation (26.4%) also prevalent. Baseline demographic and clinical characteristics of the cohort are summarized in Table 1.

Both the right atrial and the right ventricular leadless pacemaker were implanted with the initial single catheter in 100 of 102 patients (98.0%, 95% CI 93.1 to 99.8%). All 102 patients ultimately received both devices. In the two remaining patients (2.0%, 95% CI 0.2 to 6.9%), a mechanical problem with the delivery catheter arose during right ventricular implantation, which was the first stage of the procedure in every case. In the first patient the catheter had to be exchanged before the ventricular device could be released, and the ventricular implant was completed with the replacement catheter. In the second patient the ventricular device was released with the original catheter, but the catheter was exchanged before the atrial implant because of an audible warning sign. Both patients received the atrial device through the replacement catheter. No catheter malfunctioned during right atrial deployment (0 of 102, 95% CI 0 to 3.6%), although the atrial device was implanted second in every case.

In the first patient, after a second fixation attempt of the ventricular LP, the delivery-catheter handle failed to slide forward and engage the short tether mode. The handle was jammed in the docking position. The device was fully engaged to the delivery catheter and therefore prevented the device from being released. The device was successfully disengaged from the endocardium and removed. Abbott’s product analysis revealed that the release-tether wire was bent inside the handle, and the torque coil was noted to be offset distal to the transition zone (Figure 2). Dimensional analysis found all the measurements were within product specification and the X-ray examination of the entire length did not identify any issues with the handle region, or transition zone.

In the second patient, an audible clicking sound was noted during a second deployment attempt (after an initial fixation and subsequent recapture of the ventricular device), raising concern for a mechanical issue with the catheter. The RV LP was successfully deployed but the delivery catheter was exchanged prior to atrial LP implant. The X-ray analysis of the delivery catheter found the hypotube of the shaft fractured in two places (just distal to the handle and mid-shaft) and the release-tether wire bent inside the handle (Figure 2). The dimensional analysis found all the measurements were within product specification. In both cases, challenging anatomy made RV LP implantation difficult, requiring excessive torquing, deflection, redeployment and repositioning of the delivery catheters.

The mean time from sheath insertion to removal was 69 min (range 28 to 145 min). Mean fluoroscopy time was 16.0 ± 6.6 min. In the two cases that required catheter exchange, the total procedure duration was 80 min and 65 min. These are descriptive values from a single arm.

Two device dislodgements occurred in the cohort (2 of 102, 2.0%, 95% CI 0.2 to 6.9%). One was intraprocedural and one was a right atrial device that embolized the day after implantation. Neither was related to the delivery catheter. In the intraprocedural case the device was released from the catheter normally, with no difficulty at the docking or release mechanism and no evidence of catheter malfunction, so the dislodgement reflected fixation at the implant site rather than delivery-system performance. Both devices were implanted with a single catheter. No cardiac perforation, pericardial effusion or access-site complication occurred. Thirty-day follow-up was available for all 102 patients. Apart from the two dislodgements described above, no complication occurred in any patient through 30 days after implantation, so 30-day freedom from complications was 98.0% (95% CI 93.1 to 99.8%). This agrees with the separately reported follow-up of the same cohort [6]. The confidence interval around two events runs from 0.2% to 6.9%, so this series cannot exclude a complication rate several times higher than the point estimate.

Economic analysis showed that eliminating the second delivery catheter reduced average direct implant costs by the cost of the second catheter per procedure. Because the delivery catheter is the single largest disposable item unique to dual-chamber leadless implantation, omitting the second catheter in 100 of 102 cases yielded immediate savings without introducing any offsetting expenses. Staff time, room time, anesthesia, imaging, capital equipment, overhead, and downstream costs were not measured, and no time-driven activity-based costing was performed, so these figures are a direct supply cost difference.

## 4. Discussion

This retrospective series, the largest so far to examine a single-catheter technique for dual-chamber leadless pacing, shows that both devices can be delivered through one catheter in the great majority of cases. Feasibility is what the design supports. The series has no control group, and 102 patients with two device dislodgements and two catheter failures cannot establish that the technique is as safe as the two-catheter workflow or that it shortens the procedure. Freedom from complications at 30 days was 98.0%, close to the 99% reported at 30 days in the multicenter commercial experience [7] and above the 88.9% in the i2i procedural analysis [8], although complication definitions differ between these studies and the i2i figure includes arrhythmic events. A validated single-catheter technique could simplify the workflow and lower disposable cost, but confirming that will take a prospective controlled study.

Findings from this cohort have been reported in part elsewhere, and the relationship between those reports and the present one should be stated plainly. The single-catheter observation was first presented in abstract form at Heart Rhythm 2026. Procedural and electrical outcomes for the same 102 patients, with device follow-up at 1 to 3 and 3 to 6 months, are reported separately [6]. Neither of those reports examines the delivery catheter. What this full report adds is the catheter-level analysis: the rate at which a single catheter completed both implants, the two mechanical failures together with the manufacturer’s device analysis, the handling and exchange criteria we now apply between the two implants, and the supply cost calculation with its sensitivity analysis.

Delivery-system failure rates for LP across reports are low (approximately 1%) and compatible with our study but clinically important because they can lead to embolization, procedural abortion, or the need for emergency retrieval [9,10]. All delivery catheter malfunctions occurred during RV LP implant. Surprisingly, no malfunctions were observed during RA LP implant, the stage at which a malfunction might be expected and where the conventional workflow would introduce a second, fresh catheter.

The delivery catheter is composed of a torqueable shaft that couples a braided hypotube to a handle-mounted control knob, translating operator rotation into precise distal torque for docking, fixation, and retrieval with 1:1 torque delivery even through tortuous venous anatomy [4]. An integrated deflection lever with a locking brake lets the operator steer and hold the curve, while a retractable protective sleeve shields the pacemaker’s helix during vascular navigation.

### 4.1. Mechanistic Insights

Both catheter malfunctions followed the recapture and attempted redeployment of the right ventricular pacemaker. Both also occurred during ventricular implantation, before the catheter had been reloaded with the atrial device. They therefore speak to mechanical stress during a difficult ventricular implant, and not to cumulative damage from sequential use in two chambers. Mechanical fatigue of the tether or hypotube under repeated torque and traction is the most plausible explanation, but with two events, and with the manufacturer’s dimensional analysis finding all measurements within specification, this remains a hypothesis rather than a demonstrated mechanism. The number of fixation and recapture attempts was not recorded prospectively across the cohort, so catheter performance cannot be stratified by recapture count, and we cannot say whether catheters subjected to two or more recaptures behaved differently from those subjected to none. Table 2 lists handling factors that could plausibly damage the torque-transmission elements. It is derived from the device construction, the two product analyses, and the published reports of tether failure in other systems, rather than from factors observed in these two patients, and it should be read as hypothesis-generating. Blood ingress at the distal cap, for example, was not identified in either case. No catheter failed during atrial implantation, which is consistent with the delivery system remaining serviceable after reuse, though 102 cases cannot establish that. Implanting the ventricular device first, while catheter integrity is greatest, and moderating torque during the atrial implant, is the workflow these observations support.

Other reports show examples of the other LP delivery device malfunction. During a Micra™ implant, seven recapture attempts in the RV septum overstressed the delivery tether; a final forceful pull snapped the tether, leaving the device stuck in the ventricle. The pacemaker was later snared and removed, illustrating that repeated redeployments and high traction can fracture the Micra tether [11,12].

Eliminating the second catheter reduced the direct disposable supply cost by the cost of the second catheter, a saving that accrues to the implanting center because the second delivery catheter is the disposable specific to the dual-chamber leadless workflow. This is a difference in supply cost, not a cost-effectiveness result. It is consistent with work on atrial fibrillation ablation showing that operator-selected disposables account for a substantial share of procedural cost and that equipment choice is a lever the physician controls [13,14].

Procedure times in this series were similar to those of earlier AVEIR DR studies. Our mean sheath insertion to removal time was 69 min, with a mean fluoroscopy time of 16.0 ± 6.6 min. In the AVEIR DR i2i procedural analysis, across 452 patients and 126 implanters, the same interval was 90 ± 37 min, with 20 ± 13 min of fluoroscopy [8]. The multicenter commercial series reported 64 ± 33 min from initial incision to final suture, with 13 ± 8 min of fluoroscopy [7]. That interval adds only venous access and closure to ours, so the two can reasonably be set side by side. Our times are closest to those of experienced implanters. In the i2i learning-curve analysis, sheath insertion to removal time fell from 93 ± 36 min over an implanter’s first four procedures to 76 ± 34 min from the ninth procedure onward, and fluoroscopy time fell from 21 ± 13 to 14 ± 8 min [8]. Our 69 min and 16.0 min fall within that advanced-experience range, and our 30-day freedom from complications of 98.0% is in line with the 98% reported with advanced experience in the same analysis, although complication definitions differ. This fits the way the technique was introduced: our operators adopted the single-catheter approach only after the center’s first 20 dual-chamber implants, and their experience kept growing over the 102 procedures that followed. For the same reason the comparison cannot show that the single-catheter workflow is faster. These are external cohorts, and any difference is at least as likely to reflect accumulated operator experience as the change in catheter strategy. What the data do suggest is that reusing the catheter did not lengthen the procedure. The workflow is otherwise unchanged, and the only step the technique removes is the preparation of a second catheter.

The pattern here is a familiar one in pacing and device therapy. A procedural change that simplifies an implant has to be judged on safety and reintervention as well as on efficiency, and the two arguments have to be made separately. Randomized comparisons of conduction system pacing against biventricular pacing, for instance, reported functional and composite clinical gains while finding no difference in complications, procedural duration or reintervention, so the procedural case rested on its own evidence [15]. The same was true of pulsed field ablation in patients with existing cardiac implantable electronic devices, where adoption moved ahead of the device-interaction data and each question had to be answered on its own terms [16]. A single-catheter workflow should be held to that standard. This series supplies the feasibility and supply cost side of the argument. The safety and reintervention side needs a controlled study with longer follow-up.

### 4.2. Recommendations

Although our series shows that a single-catheter technique is feasible in most cases, we reserve a second, fresh delivery catheter whenever procedural conditions impose excess mechanical stress on the first system, specifically, if multiple recapture attempts were performed, if extreme torque or deep deflection is required to reach a challenging atrial or ventricular anatomy. Intra-procedurally, immediate catheter exchange is advised the moment the handle cannot transition into short or long-tether mode, when unexpected resistance or shaft kinking is seen under fluoroscopy, or when unusual clicking sounds arise from the handle during fixation which might be an early warning of hypotube fatigue, tether kinking, or impending release-mechanism failure. Proactively switching to a new catheter under these circumstances mitigates the risk of tether fracture, device embolization, or difficulty releasing the LP.

### 4.3. Limitations

Several limitations restrict what this series can support. It is retrospective and single arm. The 20 two-catheter procedures that preceded adoption of the technique were not analyzed and do not provide a usable comparator, so no internal control group exists, and every comparison with published two-catheter cohorts is subject to selection, temporal and learning-curve confounding. Because the study is single arm and has no comparator, it can characterize observed acute outcomes but cannot establish that the technique is safe or that it shortens the procedure. Five operators at one high-volume center performed the procedures, and the technique was adopted only after initial institutional experience, so the success rate may partly reflect accumulated experience rather than the catheter strategy; outcomes were not analyzed by chronological case sequence or by operator, and generalizability to less-experienced sites is uncertain. With two device dislodgements in 102 patients, and separately with two catheter failures, each 95% confidence interval runs from 0.2% to 6.9%; neither excludes a rate materially higher than the point estimate. The number of fixation and recapture attempts was not recorded prospectively, which prevents any analysis of catheter performance by recapture count, the variable most likely to matter. Finally, the economic analysis compares disposable acquisition prices at one institution in 2025. No time-driven activity-based costing was performed, and staff, room, anesthesia and overhead costs were not measured, so the 6% figure is a difference in direct supply cost and should not be read as a cost-effectiveness result.

## 5. Conclusions

In experienced hands, sequential implantation of dual-chamber Aveir leadless pacemakers through a single delivery catheter was acutely feasible. Both devices were delivered with the initial catheter in 98% of 102 consecutive patients, and the direct disposable supply cost fell by the cost of the second catheter per case. Apart from the two device dislodgements, no complication occurred through 30 days after implantation. The design is retrospective and uncontrolled and the number of events is small, so these results should be read as procedural feasibility with 30-day freedom from complications of 98.0%, together with a difference in supply cost. They do not establish that the technique is as safe as the two-catheter workflow, that it shortens the procedure, or that it should replace the two-catheter workflow described by the manufacturer. Both catheter failures followed repeated recapture and torque during ventricular implantation, and we now exchange the catheter when those conditions arise. A prospective controlled study, with longer follow-up and prospectively recorded recapture data, is needed before the approach can be recommended more widely.

## Figures and Tables

**Figure 1:**
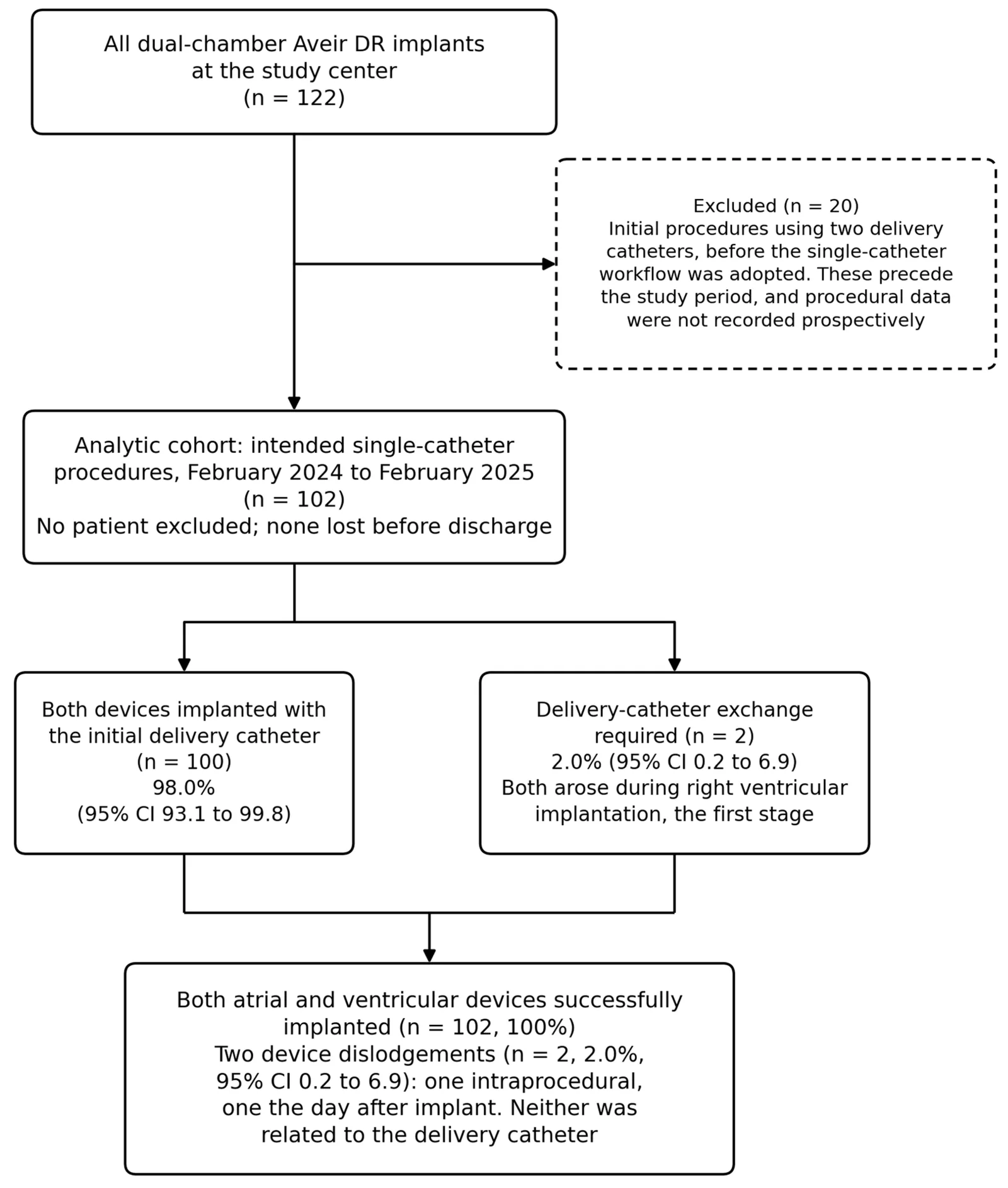
Patient flow. All dual-chamber Aveir DR implants performed at the study center, the 20 initial two-catheter procedures excluded from analysis, the 102-patient analytic cohort, and acute outcomes with 95% confidence intervals.

**Figure 2:**
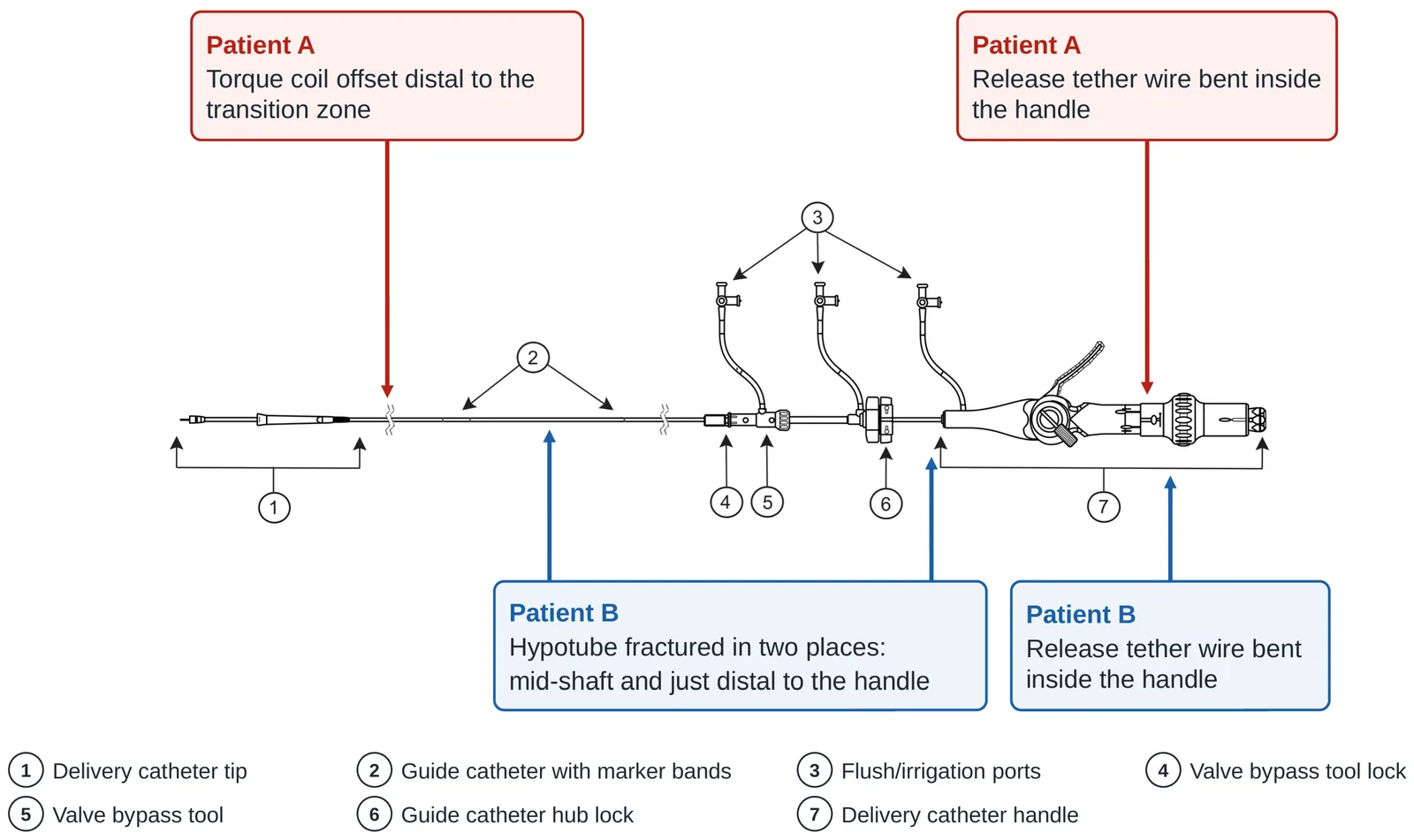
Sites of delivery-catheter malfunction in the two patients who needed a catheter exchange. The full-length, single-use Aveir™ delivery catheter is shown with its main components numbered 1 to 7, as listed in the key. Red callouts mark the findings of the manufacturer’s analysis of the catheter from Patient A, the first case described in the Results: the torque coil was offset distal to the transition zone, and the release-tether wire was bent inside the handle. Blue callouts mark the findings in Patient B, the second case: the hypotube was fractured in two places, mid-shaft and just distal to the handle, and the release-tether wire was bent inside the handle. Both malfunctions arose during right ventricular implantation, and dimensional analysis of both catheters found all measurements within product specification. Schematic adapted from the manufacturer’s instructions for use [4].

**Table 1:** Baseline characteristics.

Characteristics	(*n* = 102)
Mean age (years)	77.3 ± 10.0
Male (%)	59 (57.8)
BMI (kg/m^2^)	28.6 ± 7.2
Race (%)	
Caucasian	84 (82.4)
African American	4 (3.9)
Others	14 (13.7)
Congestive Heart Failure (%)	19 (18.6)
Hypertension (%)	84 (82.4)
Atrial fibrillation (AF) (%)	
No AF	74 (72.5)
Paroxysmal AF	19 (18.6)
Persistent AF	3 (2.9)
Permanent AF	5 (4.9)
Atrial flutter	1 (1.0)
Coronary Artery Disease (CAD) (%)	36 (35.3)
Myocardial Infarction (MI) (%)	9 (8.8)
Diabetes Mellitus (%)	43 (42.2)
Chronic Kidney Disease (%)	14 (13.7)
Cerebrovascular accident/transient ischemic attack (%)	17 (16.8)
Chronic Obstructive Pulmonary Disease (%)	9 (8.8)
Ejection Fraction (%)	59.0 ± 13.2
Indications (%)	
Sinus Node Dysfunction/Sinus Bradycardia	49 (48.0)
Tachy-Brady syndrome	13 (12.7)
Complete Heart Block	21 (20.6)
2nd Degree/High-Degree Heart Block	19 (18.6)

**Table 2:** Postulated contributing factors and mechanisms of delivery-catheter mechanical trauma. The factors listed are hypothesized from the device construction, the manufacturer’s analysis of the two failed catheters, and the published reports of delivery-system failure in other leadless systems. They were not each observed in the two patients reported here and are not established mechanisms. The table is intended to generate hypotheses and to inform careful catheter handling and timely exchange.

Contributing Factor	Mechanism of Mechanical Trauma
Multiple recapture/deployment attempts	Repeated torque and traction cycles fatigue the hypotube and tether wire, leading to micro-cracks and eventual break
Torquing in confined or tortuous anatomy	Sharp bends concentrate stress on the braided hypotube and torque coil, increasing risk of kinking or fracture
Re-docking after partial deployment	Engaging and disengaging the docking mechanism under tension can kink or fray the tether bullets and wire
Excessive shaft bending or deflection	Over-deflection beyond design limits compromises torque transmission integrity and predisposes the hypotube to break
Rotation with the shaft under tension	Twisting the control knob when the catheter is curved concentrates stress at bend points, risking hypotube fracture
Ignoring resistance or unusual clicking in handle	Continuing manipulation despite audible/tactile warning cues propagates existing micro-damage to catastrophic failure
Blood ingress at distal cap	Entrapped blood clots increase friction in the tether channel and mask early signs of mechanical binding

## Data Availability

The data presented in this study are available on request from the corresponding author due to (specify the reason for the restriction).
